# Motor oscillations reveal new correlates of error processing in the human brain

**DOI:** 10.1038/s41598-024-56223-x

**Published:** 2024-03-07

**Authors:** Juliana Yordanova, Michael Falkenstein, Vasil Kolev

**Affiliations:** 1 Institute of Neurobiology, Bulgarian Academy of Sciences, Acad. G. Bonchev str., bl. 23, 1113 Sofia, Bulgaria; 2 Institute for Working Learning Ageing (ALA Institute DE), Bochum, Germany

**Keywords:** Response-related potentials, EEG, Brain oscillations, Theta/delta, Error processing, Performance monitoring, Cognitive control, Cognitive neuroscience, Motor control, Sensorimotor processing

## Abstract

It has been demonstrated that during motor responses, the activation of the motor cortical regions emerges in close association with the activation of the medial frontal cortex implicated with performance monitoring and cognitive control. The present study explored the oscillatory neurodynamics of response-related potentials during correct and error responses to test the hypothesis that such continuous communication would modify the characteristics of motor potentials during performance errors. Electroencephalogram (EEG) was recorded at 64 electrodes in a four-choice reaction task and response-related potentials (RRPs) of correct and error responses were analysed. Oscillatory RRP components at extended motor areas were analysed in the theta (3.5–7 Hz) and delta (1–3 Hz) frequency bands with respect to power, temporal synchronization (phase-locking factor, PLF), and spatial synchronization (phase-locking value, PLV). Major results demonstrated that motor oscillations differed between correct and error responses. Error-related changes (1) were frequency-specific, engaging delta and theta frequency bands, (2) emerged already before response production, and (3) had specific regional topographies at posterior sensorimotor and anterior (premotor and medial frontal) areas. Specifically, the connectedness of motor and sensorimotor areas contra-lateral to the response supported by delta networks was substantially reduced during errors. Also, there was an error-related suppression of the phase stability of delta and theta oscillations at these areas. This synchronization reduction was accompanied by increased temporal synchronization of motor theta oscillations at bi-lateral premotor regions and by two distinctive error-related effects at medial frontal regions: (1) a focused fronto-central enhancement of theta power and (2) a separable enhancement of the temporal synchronization of delta oscillations with a localized medial frontal focus. Together, these observations indicate that the electrophysiological signatures of performance errors are not limited to the medial frontal signals, but they also involve the dynamics of oscillatory motor networks at extended cortical regions generating the movement. Also, they provide a more detailed picture of the medial frontal processes activated in relation to error processing.

## Introduction

To achieve a goal, humans have to build a neural plan of the targeted goal, maintain this plan in working memory, and compare the result of the behaviour with the intended action^1,2^. Therefore, a continuous matching of the planned action with the actual one occurs in the brain, known as performance monitoring. Performance monitoring is recognized as a crucial component of the executive control system.

An advantageous step for investigating performance monitoring was the discovery of a negative brain potential, error negativity (Ne)^3^, also termed error-related negativity (ERN)^4^. The Ne is elicited or substantially enhanced by performance errors at medial fronto-central regions, peaking at around 80–100 ms after the error^3,5^. Source analyses have localized the generator(s) of Ne in the medial frontal cortex engaging the anterior cingulate cortex (ACC) and the pre-supplementary/supplementary motor area (pre-SMA/SMA)^5,6^, both of which have been strongly implicated with behavioural adaptation and control (e.g. Refs.^1,5,7^). Initially, the Ne has been associated with detecting a mismatch between the intended and actual movement^8,9^. Different models have been subsequently proposed for the functional correlates of Ne, e.g. the amount of conflict between concurrent response options^7,10,11^, detecting behavioural outcomes that are unexpected^12^, worse than expected^13^, or disconfirming a prediction^14^. All existing models recognize the Ne as a fundamental neurophysiological marker of error processing and performance monitoring in the human brain.

The error negativity is traditionally computed and analysed in the time domain. According to the concept of event-related oscillations (e.g. Ref.^15^), time-domain potentials emerge from the event-related reorganization of ongoing electroencephalographic (EEG) rhythms and are composed of multiple frequency-specific responses. Time–frequency decomposition has demonstrated that the Ne is a heterogeneous signal composed of at least two major sub-components from delta (1–3 Hz) and theta (3.5–7 Hz) frequency bands, differentially associated with performance and movement monitoring^16^. Notably, it has been found that response-related potentials (RRPs) generated by movements at motor regions also contain delta and theta EEG activity^16–20^. Response-related time–frequency components are phase-locked by the movement and are pronounced at motor cortical regions of the hemisphere contralateral to the response, not being well expressed at motor regions ipsilateral to the response^17,18,21^.

These observations show that similar frequency-specific components characterize the error negativity and motor potentials generated by movements. Moreover, during response production, the activation of the motor cortical regions emerges in close association with the activation of the medial frontal cortex. Urbano et al.^22,23^ have demonstrated that during both uni- and bilateral voluntary movements, the premotor, motor, and sensorimotor cortical regions contra-lateral to the movement (M1-S1) and the medial frontal regions (SMA) are co-activated. The functional coupling between M1-S1 and SMA persists during all phases of movement production – preparation, initiation, and execution implying the existence of continuous communication between the two areas. Notably, this inter-regional coupling is supported by oscillatory networks from delta (1–3 Hz) and theta (4–7 Hz) frequency ranges^23^. For theta, alternating phase-reversal patterns have been demonstrated between the contra-lateral M1-S1 and the SMA regions for voluntary movements^23^, sensorimotor responses, and errors^16,19,20,24^, suggesting that a cross-talk mechanism^23^ is reinforced by theta networks. Indeed, according to Duprez et al.^25^, a phase-synchronized theta network with a “hub” in the medial fronto-central cortex^26,27^ supports various executive functions by cyclically orchestrating brain computations related to the cognitive control of response execution in different contexts.

These observations imply that neural correlates of error processing may be present at both the medial frontal regions as reflected by Ne and motor cortical regions. It can be hypothesized that a dysregulated coordination of the motor cortex due to a wrong or a conflicting command, or continuous feedback from frontal regions signalling about an emerging error would modify the characteristics of motor potentials. It can be therefore expected that the oscillatory neurodynamics of motor potentials would differ between correct and error responses. However, the major focus of error processing research has remained on the evaluative functions of the medial frontal signal Ne^5,6^, while motor cortical potentials have not been studied systematically in this context. Hence, the objective of the present study was to explore the oscillatory neurodynamics of response-related potentials generated by performance errors in a sensorimotor task.

For that aim, response-related potentials of correct and incorrect responses were explored in a sample of young adults who performed a choice reaction sensorimotor task. Oscillatory activity generated at premotor, motor, and sensorimotor cortical regions contra- and ipsilateral to the responding hand was analysed with respect to power, and temporal and spatial synchronization of frequency RRP components. Since the power and temporal synchronization of delta and theta oscillations at medial frontal areas have manifested an increase during errors^16,28^, these parameters were computed to explore if RRPs also are sensitive to error processing in a similar way. Spatial synchronization was analysed within the hypothesis that the communications of motor generation regions are altered. It was expected that the connections of motor-generation areas would be suppressed or re-localized during errors due to a dysregulated coordination of the motor cortex. In view of the central role of ACC/SMA in performance monitoring^5,6^, the synchronization between medial frontal and motor-generation regions was specifically targeted.

## Methods

### Participants

A total of 16 young adults were studied. All participants were healthy, without a history of neurologic, psychiatric, chronic somatic, or hearing problems. They were under no medication during the experimental sessions, with normal or corrected-to-normal vision. This sample size was reduced due to the insufficient number of artefact-free error trials in 6 of the subjects (exclusion criteria are presented in detail in 2.4.) and included 10 young adults (5f, mean age 22.5 years, SE ± 1.5). The study received approval from the Research Ethics Committee at the Leibniz Institute for Working Environment and Human Factors, Dortmund, Germany. Prior to engaging with the study, all participants gave informed consent in line with the Declaration of Helsinki.

### Task

A four-choice reaction task (CRT) was employed as reported in Yordanova et al.^16^. Four stimulus types represented by the letters A, E, I, and O were delivered randomly with an equal probability of 25% in separate experimental blocks. A total of 200 stimuli were presented in each block, with n = 50 for each stimulus type. The letters A, E, I, and O had to be responded to with the left middle, left index, right index, and right middle fingers, respectively. They were designated as four stimulus–response (SR) types (SR1, SR2, SR3, and SR4). Response force was measured by sensometric tensors while subjects produced a flexion with each of the four fingers. Subjects performed the CRT in two modalities—auditory and visual. Auditory stimuli (duration 300 ms, intensity 67 dB SPL) were delivered via headphones binaurally, with similar envelopes of the sound pressure waves formed for all stimuli. Visual stimuli with the same duration were shown in the middle of a monitor placed 1.5 m in front of the subject’s face. Inter-stimulus intervals varied randomly between 1440 and 2160 ms (mean 1800 ms). To keep time pressure, a feedback tone was delivered at 700 ms after stimulus onset if the response was longer than this threshold. This tone had to be avoided by responding fast enough. No feedback about response accuracy was provided. The participants had a short training session to memorize the stimulus–response types. A total of nine auditory and nine visual CRT blocks were performed by each subject. Sequences of auditory and visual blocks were counterbalanced across participants.

### Data recording and processing

Data from all nine blocks in each modality were used. EEG was recorded from 64 channels with Cz as reference, with frequency limits of 0.1–70 Hz, and a sampling rate of 250 Hz. EEG traces were visually inspected for gross electrooculogram (EOG) and electromyogram (EMG) artefacts. Mechanograms from each finger were recorded to provide for analysis of motion dynamics, characteristics, and correctness. EMG from the responding hands also was registered. Contaminated trials were discarded along with EEG traces exceeding ± 100 µV. Slight horizontal and vertical eye movements preserved in the accepted trials were corrected by means of a linear regression method for EOG correction^29^. Data processing was performed using Brain Vision Analyzer 2.2.2 (Brain Products GmbH, Gilching, Germany).

### Response-related potentials

Response-related potentials were computed with a trigger corresponding to a threshold level of 5 N in the mechanogram. This threshold was chosen to enable a precise distinction between full and partial errors and include only full errors in the analysis. Initial observation of data indicated that on many trials, initiated incorrect responses were quickly disrupted and also could be followed by a corrective response. The force of such partial errors did not reach 5 N, in contrast to the observed force of correct responses and full errors, which determined the currently applied threshold. In this way, incomplete responses were disregarded. Although this trigger did not fully coincide with the peak of the EMG that appeared 20–40 ms earlier, it was used to justify the lack of differences in the mechanic properties of correct and error movements. In addition, this trigger warranties a more reliable elicitation of pure RRPs by avoiding contamination with overlapping activations (e.g. simultaneous auditory and somatosensory potentials that may accompany the commonly used keyboard button press). From a methodological point of view, the distortion of RRPs caused by preceding or overlapping stimulus-related components is to be accounted for. Adjusted filtering at the level of single EEG trials has demonstrated that such effects are relatively small for time windows preceding or coinciding with response preparation and execution^30^. On the basis of these results, RRPs were not corrected for stimulus-related components. The number of trials accepted for analysis depended on the amount of EEG artefacts, the selection of only full errors and the exclusion of slow responses (< 5%) for which a feedback was delivered. Following these criteria, for each of the four stimulus–response types (SR1, SR2, SR3, and SR4), between 20 and 30 artefact-free error trials from each individual were collected. To equalize the number of error and correct trials, for each individual and SR type the same number of artefact-free correct trials were included following a randomized inclusion procedure. Responses with left-hand fingers (SR1 and SR2) were combined and responses with right-hand fingers (SR3 and SR4) also were combined to produce RRPs for left- and right-hand responses separately. Thus, between 40 and 60 single trials for each participant in each modality (auditory and visual) for each hand (left and right) were used for RRP analysis of correct and incorrect responses. In a variety of previous studies of error processing, a small number of EEG error trials was accepted as adequate for analysis (e.g. Ref.^31^). However, we considered it important to account for the signal-to-noise ratio in the EEG signals by using a sufficiently high and equal number of trials for correct and error responses. This stringent control of the number of artefact-free error trials lead to the exclusion of 6 participants from the original sample. It is important to emphasize that the error rate was not smaller in the excluded participants (p > 0.1) so that the results were not biased by performance quality. Details of RRP analysis in the time- and time–frequency domains described in the following are presented in Yordanova et al.^16,18^.

### Current source density

To achieve a reference-free evaluation, all data analyses were performed after current source density (CSD) transform of the signals (e.g. Refs.^32–35^). The exact mathematical procedure is presented in detail in Ref.^34^. The algorithm applies the spherical Laplace operator to the potential distribution on the surface of the head. The CSD transform replaces the potential at each electrode with the current source density of the electrical field calculated from all neighbour electrodes, thus eliminating the reference potential. When applied with dense electrode arrays (48–256 electrodes, 64 in the present study), this procedure provides excellent estimates of the bioelectric activity of the cortical surface^36^. For all analyses, CSD-transformed RRPs were used.

### Time-domain analysis

The epoch for RRP analysis in the time-domain had a length of 1600 ms. The moment of response production was positioned in the centre of this epoch corresponding to 5 N in the mechanogram. A baseline of 800–600 ms before the response was chosen such as to precede stimulus delivery and avoid contamination by stimulus-related potentials and processing. RRP components in the time domain were identified to verify the motor-related signals and their topographic distribution. However, as being out of the scope the present research, they were not extensively analysed.

### Time–frequency decomposition

Time–frequency (TF) analysis of RRPs was performed by means of a continuous wavelet transform (CWT, details in Ref.^16^). Wavelets *W(t,f)* can be generated in the time domain for different frequencies, *f*, according to the equation:

$$W\left( {t,f} \right) = A\exp \left( { - t^{2} /2\sigma t^{2} } \right)\exp \left( {2i\pi ft} \right),$$

where *t* is time,$A = \left( {\sigma_{t} \sqrt \pi } \right)^{ - 1/2}$, *σ*_*t*_ is the wavelet duration, and $i = \sqrt { - 1}$. For this analysis, wavelet family was characterized by a ratio of *f*_*0*_/*σ*_*f*_ = 4, where *f*_*0*_ is the central frequency and *σ*_*f*_ is the width of the Gaussian shape in the frequency domain. The choice of the ratio *f*_*0*_/*σ*_*f*_ was oriented to the expected slower phase-locked components present in the response-related potentials, which had an effect on the shape of the Morlet wavelet and decreased its decay^16,18^. The analysis was performed in the frequency range of 0.1–16 Hz with a central frequency at 0.4 Hz intervals. For different *f*_*0*_, time and frequency resolutions can be calculated as 2* σ*_*t*_ and 2* σ*_*f*_ , respectively^37^. *σ*_*t*_ and *σ*_*f*_ are related by the equation *σ*_*t*_ = 1/(2π*σ*_*f*_).

To achieve a reliable analysis of low-frequency components in the time–frequency domain and avoid possible edge effects, 4096 ms-long epochs were used for RRPs, with the moment of response execution (5 N) being in the centre of the analysis epoch. TF decomposition was performed on CSD-transformed single-trial RRPs. Based on observations of grand averages for all TF parameters used here (see “Parameters”) CWT layers corresponding to theta range (*f*_*0*_ = 5.5 Hz) and delta range (*f*_*0*_ = 1.9 Hz) were used for subsequent analyses. Delta and theta layers were extracted as representing relevant frequency ranges of RRPs (see Fig. 3).

### Total power

Total power (TOTP) comprises the phase-locked and non-phase-locked fractions of the signal. It was measured to represent the total energy of response-related oscillations. For each trial, the time-varying power in relevant frequency bands (delta and theta) was calculated by squaring the absolute value of the convolution of the signal with the complex wavelet.

### Temporal synchronization

The phase synchronization across trials was measured by means of the phase-locking factor (PLF, e.g. Refs.^37,38^). The PLF provides a measure of between-trial phase synchronization of oscillatory activity independently of the signal’s amplitude. The values of PLF yield a number between 0 and 1 determining the degree of between-trial phase-locking, where 1 indicates perfect phase alignment across trials and values close to 0 reflect the highest phase variability. PLF was computed for delta and theta TF components of RRPs.

### Spatial synchronization

Following methodological recommendations^39^, the phase-locking value (PLV) was used because it is robust to time dynamics, time lag, frequency mismatches, and frequency non-stationarities, as expected for response-related responses. Also, it is robust to increased variance in phase stability, as expected for errors and is recommended for hypothesis-driven analysis.

PLV measures the extent to which oscillation phase angle differences between electrodes are consistent over trials at each time/frequency point (e.g. Ref.^40^). As a measure of spatial synchronization, PLVs were computed for delta and theta TF scales at each time-point *t* and trial *j* according to the equation:

$$PLV_{k,l} = \left| {\frac{1}{N}\sum {e^{{i\left( {\rho_{j,k} \left( {t,f_{0} } \right) - \rho_{j,i} \left( {t,f_{0} } \right)} \right)}} } } \right|,$$

where *N* is the number of single trials, *k* and *l* are indices for the pair of electrodes to be compared, and *ρ* is the instantaneous phase of the signal. *PLV*_*k,l*_ results in real values between one (constant phase difference) and zero (random phase difference). PLV computation followed the approach described in Ref.^41^.

For PLV analysis 35 electrodes were used (F3, Fz, F4, FC5, FC3, FC1, FCz, FC2, FC4, FC6, T7, C5, C3, C1, Cz, C2, C4, C6, T8, CP5, CP3, CP1, CPz, CP2, CP4, CP6, P3, Pz, P4, PO5, POz, PO6, O1, Oz, and O2). PLV was computed for each pair of electrodes, resulting in a total of 595 pairs for each subject, modality, hand (left and right), and response type (correct and error).

### Parameters

As described above, all TF parameters were computed from an analysis epoch of 4096 ms duration, with the moment of response execution (5 N) being in the centre of the epoch. TF parameters were measured after delta and theta scales were extracted. An epoch from 600 to 800 ms prior to the response was used as a baseline. The single-trial mean value of this baseline epoch was subtracted from TF measures at each time point of the analysis epoch for each frequency band and electrode.

The following TF parameters were computed: TOTP and PLF at 64 electrodes, and PLV of 595 electrode pairs for two frequency bands – delta and theta. In addition, two other parameters were introduced based on the pair-wise PLV measures. To identify regions with maximal connectedness with all other cortical regions during response production, the mean of all pairs (n = 34) was computed for every single electrode, termed regional PLV (R-PLV). Also, a separate analysis used PLV measures of pairs guided by the medial fronto-central electrode FCz (FCz-PLV). This measure aimed at specifically assessing the connectivity of response monitoring regions during correct and error response generation.

For all TF parameters (TOTP, PLF, R-PLV, and FCz-PLV) the maximal value was identified in the latency range of -300 to + 300 ms around the moment of response execution for both delta and theta TF components. The parameter was measured as the mean magnitude value within -24 to + 24 ms around the maximum. In addition, the peak latency of each signal (TOTP, PLF, R-PLV, FCz-PLV) maximum was measured. For statistical evaluation, measures of TOTP were log10-transformed. These TF parameters were measured in each subject for each frequency band (delta and theta), each electrode, each hand (right and left), and each condition (correct and error).

### Statistical analyses

With regard to the possibilities that error processing may be functionally asymmetric for the right and the left hand^16^ or motor oscillations may differ depending on the differential involvement of the two hemispheres in cognitive processes or on factors such as right-hand dominance^16,42,43^, response-related activity was analysed for each hand separately at motor cortical areas contra-lateral and ipsi-lateral to the response. Fronto-central (FC), central (C), and centro-parietal (CP) electrodes were used to approximate the activity at premotor, motor/sensorimotor and extended sensorimotor regions, respectively^44,45^. The topographic localization of the effects was supported by using the spatially enhanced CSD transformed RRPs. For TOTP, PLF and R-PLV, a repeated-measures ANOVA design was applied with within-subjects variables Accuracy (Correct vs. Error) and Modality (Auditory vs. Visual). Additional within-subjects factors were included to analyse topographic effects – Region (fronto-central FC3/FCz/FC4 vs. central C3/Cz/C4 vs. centro-parietal CP3/CPz/CP4) and Laterality (left hemisphere FC3/C3/CP3 vs. midline FCz/Cz/CPz vs. right hemisphere FC4/C4/CP4), as indicated in Fig. 2. Only for R-PLV the Laterality variable included two levels – left hemisphere and right hemisphere. Peak latency values of the four TF parameters were evaluated using the same statistical designs. Whenever observed, significant interactions were explored. With regard to the focus of the study, only interactions of Accuracy with other variables are reported. The complex Accuracy x Region x Laterality interactions were investigated by exploring the Accuracy effect at each single electrode. Only significant statistical outcomes are presented in the results. Degrees of freedom of factors with more than two levels were corrected with the Greenhouse–Geisser method. Original *df* and corrected *p*-values, effect size (ŋ^2^) and mean group values ± standard error (SE) are reported. Correct and incorrect reaction times (RT) of right- and left hand responses as well as error rates were analysed in an Accuracy x Modality x Response Side (Left hand vs. Right hand) ANOVA design.

## Results

### Performance

**Figure 1:**
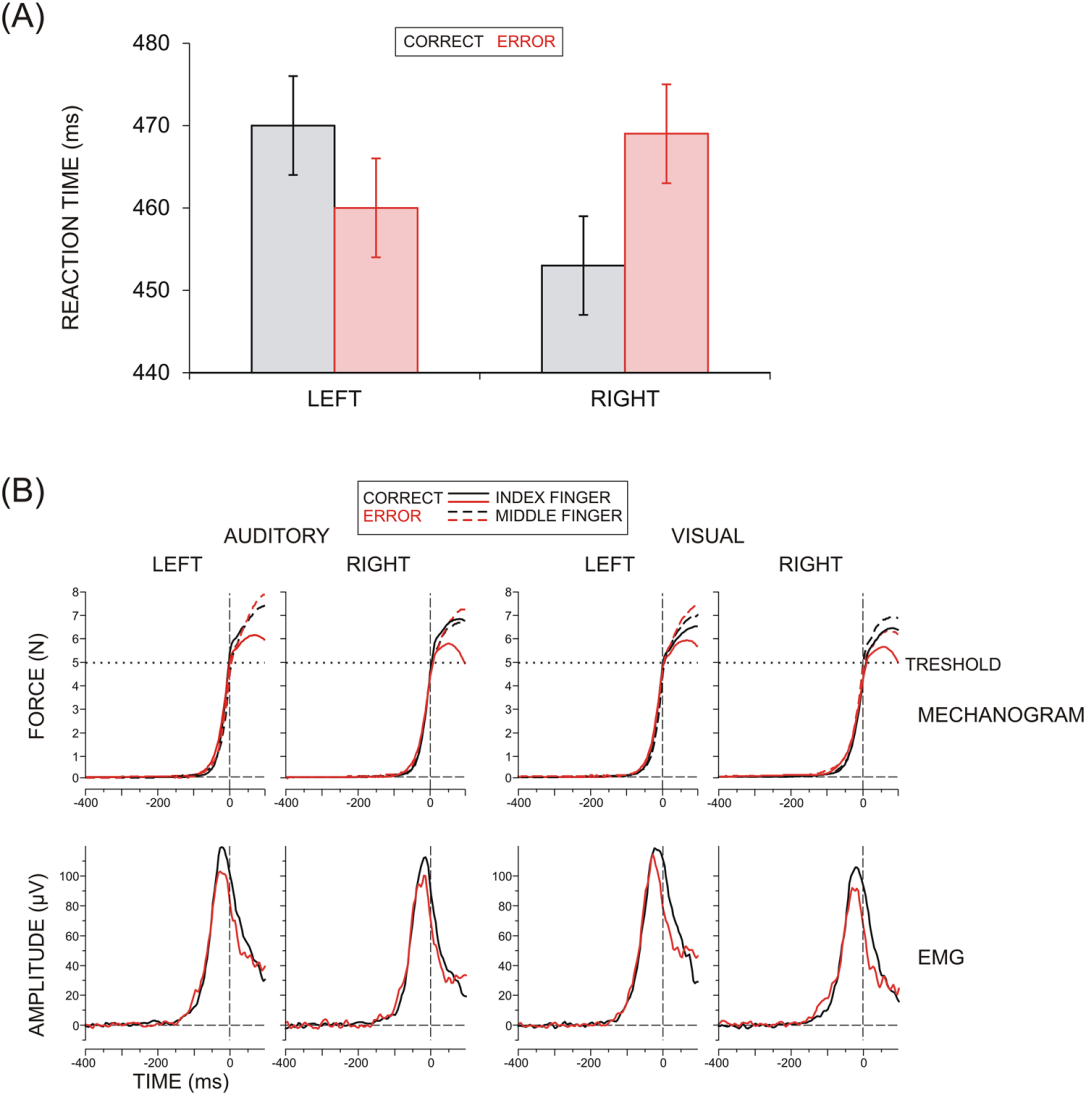
(**A**) Group mean reaction times ± SE for correct and error responses produced with the left and the right hand. (**B**) Group average mechanograms (upper panel) and electromyograms (EMG, lower panel) of correct and error responses produced with the index and middle fingers of the left and the right hand in two experimental conditions (auditory and visual). A threshold of the mechanogram at 5 N is used to determine movement onset (at 0 ms).

The main effects of Modality, Accuracy, and Side on RT were not significant (F(1/9) < 1.73, p > 0.2). As demonstrated in Fig. 1A, there was, however, a highly significant Accuracy x Side interaction (F(1/9) = 69.7, p < 0.001, ŋ^2^ = 0.875). Consistent with the right-handedness of the subjects, RTs were faster for correct right- than left-hand responses (453 ± 10.2 ms vs. 471 ± 10.1 ms; Side, F(1/9) = 8.9, p = 0.01, ŋ^2^ = 0.556). For the faster right hand, reactions were slower for error than correct responses (468 ± 12.1 ms vs. 453 ± 10.2 ms, p = 0.04, ŋ^2^ = 0.348), whereas, for the slower left hand, reactions were faster for error than correct responses (460 ± 11.2 ms vs. 471 ± 10.1 ms, p = 0.05, ŋ^2^ = 0.282) – Fig. 1A. This effect was more prominent for the visual than auditory modality (Side x Accuracy x Modality, F(1/9) = 7.8, p = 0.02, ŋ^2^ = 0.440). Error rate (mean 4.6%) did not depend on Side or Modality (p > 0.2). Figure 1B illustrates the mechanograms and electromyograms of correct and error responses of each hand for each modality. It is shown that these signals did not differ between correct and incorrect responses before the moment of movement onset or only marginal effects were seen for the EMG.

### Time-domain RRPs

**Figure 2:**
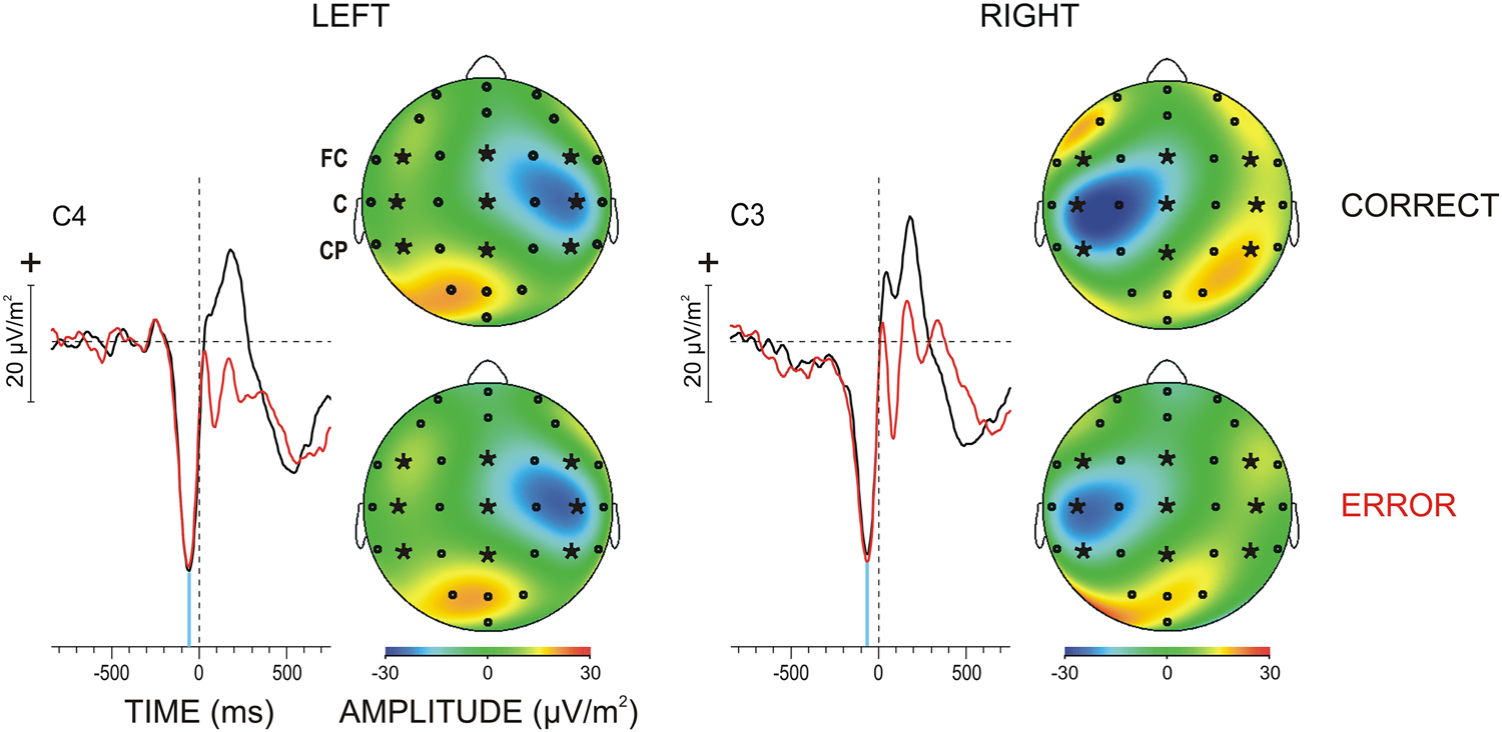
CSD transformed time-domain grand average RRPs for CORRECT and ERROR responses at motor electrodes contra-lateral to the responding hand, C4 and C3. LEFT—left-hand responses; RIGHT—right-hand responses; Response onset at 0 ms. Topography maps of the peak of pre-response negative RRP component (designated by the blue vertical line) are illustrated. The fronto-central (FC), central (C), and centro-parietal regions (CP) used for analysis are designated, with the included electrodes in the left, midline and right areas being marked by asterisks.

Figure 2 demonstrates that CSD-transformed RRPs were characterized by a negative component peaking before the response (group mean peak latency = − 60 ± 9.2 ms) and positive/negative deflections after the response. The negative pre-response component did not differ in magnitude (F(1/9) < 0.9, p > 0.7) and manifested similar topographic distributions for correct and incorrect responses of each hand (Fig. 2). Error-related differences were only observed for RRP components after the response (not evaluated here).

### Time–frequency components of RRPs

**Figure 3:**
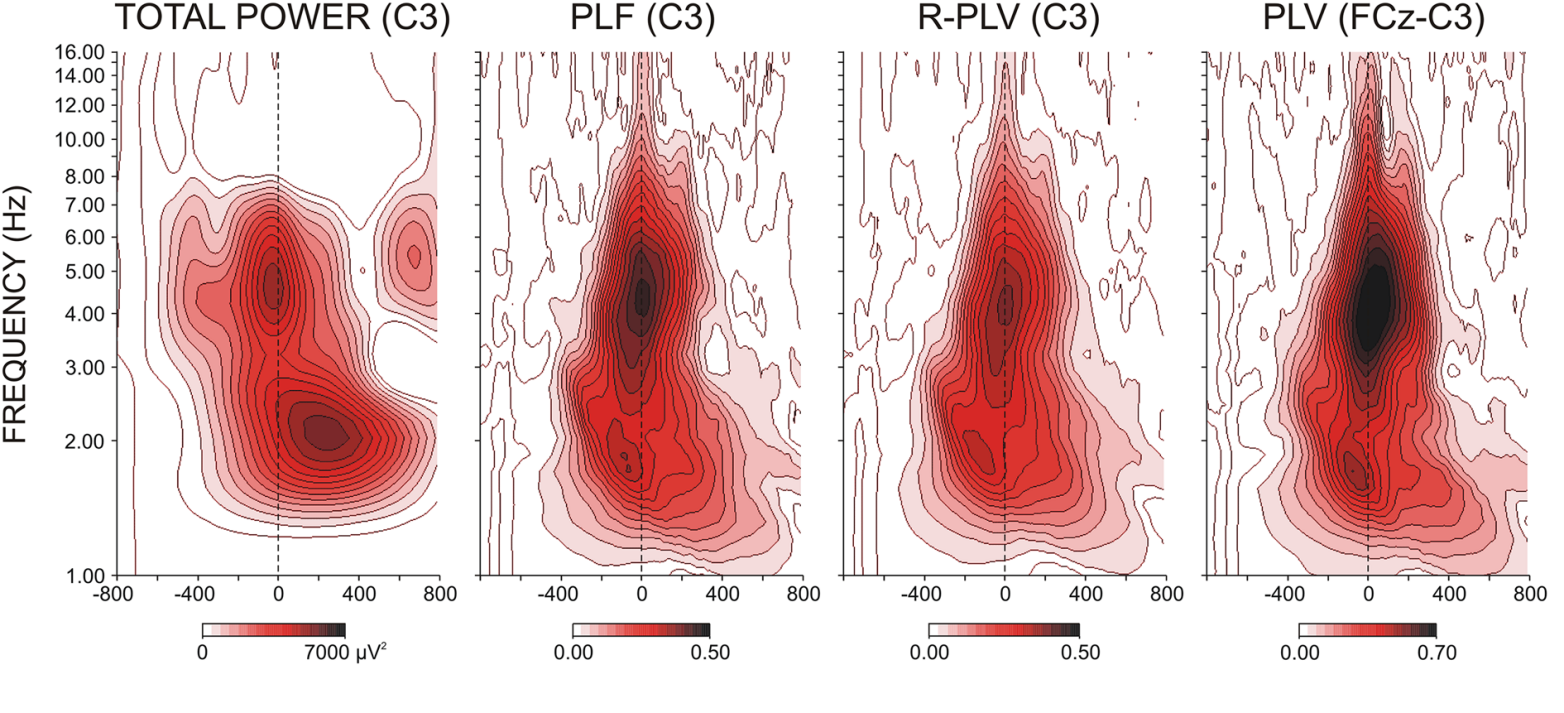
Time–frequency decomposition plots of TOTAL POWER, temporal synchronization (PLF), region-specific connectedness (R-PLV), and FCz-guided spatial synchronization (PLV) for right-hand RRPs at the contra-lateral C3 electrode. Response onset at 0 ms. Delta (1–3 Hz) and theta (3.5–7 Hz) TF components are demonstrated.

Figure 3 demonstrates that for each parameter (TOTP, PLF, R-PLV, and FCz-PLV), motor-related activity was characterized by two major TF components—delta (1–3 Hz) and theta (3.5–7 Hz). Accordingly, as detailed in the Methods, TF scales with central frequencies at 1.9 Hz and 5.5 Hz were extracted and further analysed. Figure 3 also shows that the timing of delta and theta TF components differed with respect to onset, peak, and duration relative to the moment of the response.

#### THETA

##### Theta TOTP (Fig. 4A)

**Figure 4:**
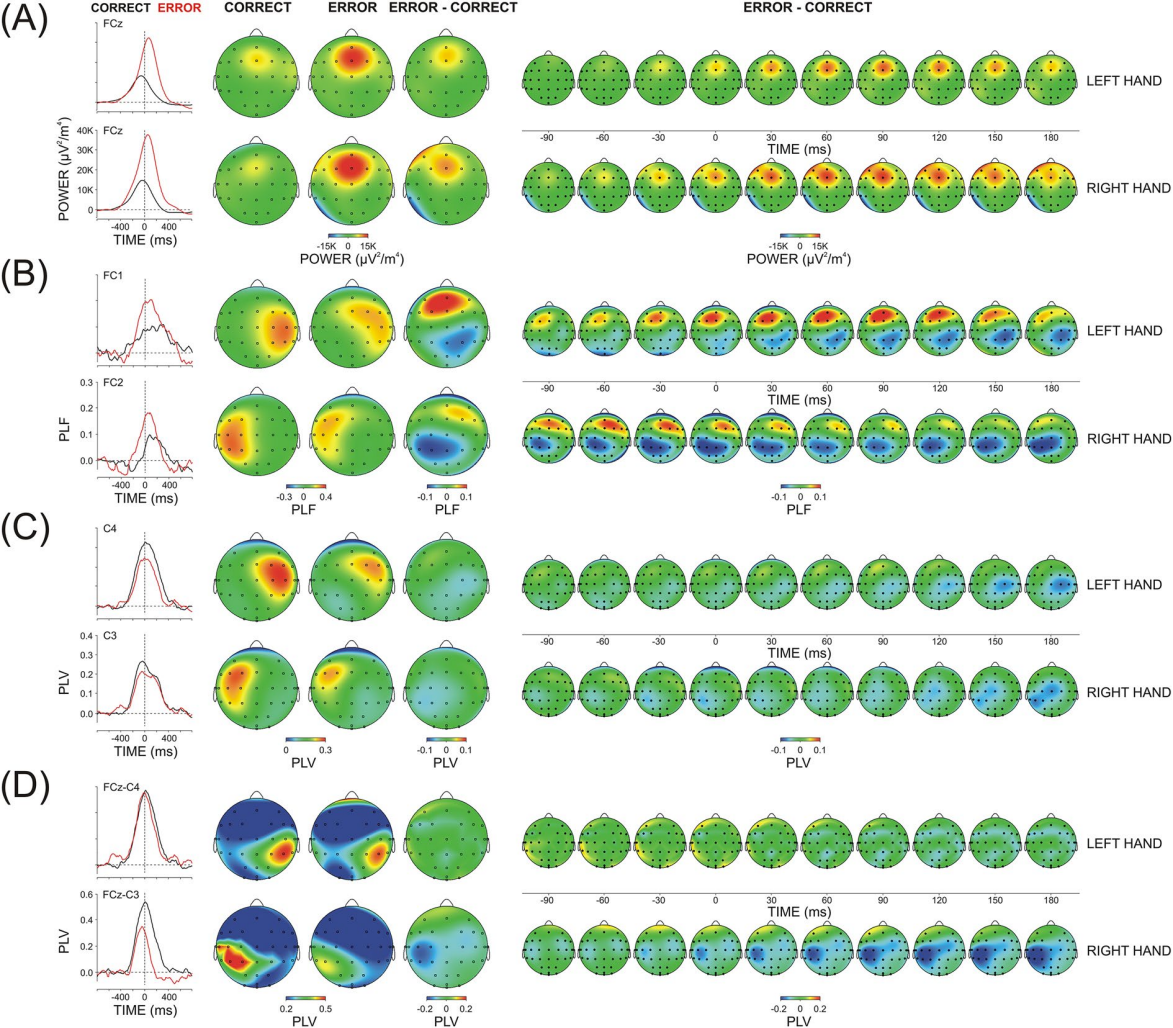
Theta TF component (3.5–7 Hz) of response-related correct and error potentials elicited by left- and right-hand motor responses (**A**) Total power, (**B**) Temporal synchronization (PLF), (**C**) Region-specific connectedness (regional PLV), (**D**) FCz-guided synchronization FCz-PLV. Left panel—Extracted theta scales at relevant mid-line, contra-, and ipsi-lateral electrodes (explained in the text); Middle panel – Topography maps for correct, error and error minus correct difference at the time of maximal expression (peak) of the signal at contra-lateral central electrodes; Right panel—Dynamic topography difference maps (error minus correct). Response onset at 0 ms.

Theta TOTP was focused on the midline fronto-central region for both the correct and error responses (Region, F(2/18) = 19.6/32.2, p < 0.001, ŋ^2^ = 0.708/0.782; Laterality, F(2/18) = 6.2/6.9, p = 0.01, ŋ^2^ = 0.428/0.478; Region x Laterality, F(4/36) = 9.4/6.3, p = 0.001/0.01, ŋ^2^ = 0.446/0.436 for left-/right-hand responses, respectively).

For errors, there was a substantial TOTP increase (Accuracy, F(1/9) = 56.3/15.4, p = 0.0001/0.004, ŋ^2^ = 0.872/0.630), which was, however, exclusively pronounced at the midline fronto-central electrode FCz (Region x Accuracy F(2/18) = 12.3/12.1, p = 0.001, ŋ^2^ = 0.566/0.574; Laterality x Accuracy, F(2/18) = 3.8/13.7, p = 0.05/ < 0.001, ŋ^2^ = 0.260/0.701; Region x Laterality x Accuracy, F(4/36) = 7.4/3.4, p = 0.003/0.04, ŋ^2^ = 0.574/0.302). Testing simple Accuracy effects at single electrodes confirmed the error-related power increase at frontal-central and central electrodes (F(1/9) = 5.1–20.1, p = 0.05–0.001, ŋ^2^ = 0.365–0.714), which was most expressed at FCz (F(1/9) = 36.8, p < 0.0001, ŋ^2^ = 0.804).

Errors were associated with different timing of the maximal expression of theta TOTP (Accuracy, F(1/9) = 69.5/54.3, p < 0.0001, ŋ^2^ = 0.882/0.867) because the peak of theta TOTP was before the response for correct responses (− 85 ± 14.3 ms), whereas it was after the response for errors (18 ± 11.6 ms) – Fig. 4A (left panel).

##### Theta PLF (Fig. 4B)

Theta PLF was significantly larger at contra- than ipsilateral regions (Laterality, F(2/18) = 29.8/26.5, p < 0.0001, ŋ^2^ = 0.768/0.746 for left-and right-hand responses, respectively).

Errors were associated with an increase of PLF at fronto-central regions accompanied by a decrease at central/centro-parietal regions (Accuracy x Region, F(2/18) = 4.01/3.9, p = 0.05, ŋ^2^ = 0.291/286). Yet, the significant interaction mainly stemmed from an inter-regional redistribution because PLF changes at fronto-central regions were modest (Accuracy, (F(1/9) = 5.0/5.1, p = 0.05, ŋ^2^ = 0.301/0.297), and did not reach significance at central and centro-parietal areas (p > 0.2). As dynamic maps imply, the fronto-central PLF enhancement involved also the ipsi-lateral hemisphere, whereas the sensorimotor PLF reduction involved mainly the contra-lateral hemisphere. In accordance with the lack of a significant Accuracy x Region × Laterality interaction, this error-related topographic re-distribution did not induce significant effects at single electrodes (F(1/9) < 4.9, p > 0.05).

The maximum of temporal synchronization occurred after the response and was significantly earlier for errors (Accuracy, F(1/9) = 11.4/8.5, p = 0.007/0.01, ŋ^2^ = 0.761/0.564; group mean of theta PLF peak latency = 61 ± 11.4 ms for correct, and 13 ± 7.5 ms for error responses) – Fig. 4B (left panel).

##### Theta R-PLV (Fig. 4C)

During both correct and error responses, region-specific connectedness with all other cortical areas was significantly stronger for the hemisphere contralateral to the response (Laterality, F(2/18) = 36.3/22.9, p < 0.001, ŋ^2^ = 0.801/0.756), with fronto-central regions being most strongly connected with other cortical regions (Region, F(2/18) = 7.1/3.9, p = 0.02/0.05, ŋ^2^ = 0.442/0.374).

Theta R-PLV did not depend significantly on whether the generated response was correct or incorrect (main and interactive Accuracy effect for each response side, p > 0.05). The maximal expression of R-PLV was after the response and occurred earlier after error (26 ± 9.6 ms) than after correct responses (49.9 ± 6.7 ms; Accuracy F(1/9) = 5.9/5.4, p = 0.04/0.05, ŋ^2^ = 0.286/0.293).

##### Theta FCz-PLV (Fig. 4D)

During both correct and error responses, theta activity at FCz electrode was most strongly synchronized with central and centro-parietal regions of the hemisphere contralateral to the response (Region, F(2/18 = 22.1/12.2, p < 0.001, ŋ^2^ = 0.789/0.679; Laterality, F(1/9) = 46.7/28.5, p < 0.001, ŋ^2^ = 0.886/0.802; Region x Laterality, F(2/18) = 5.04/6.9, p = 0.03/0.006, ŋ^2^ = 0.437/0.522).

Only for the right hand were errors associated with a reduction of FCz-guided theta synchronization at central/centro-parietal electrodes of the contra-lateral left hemisphere (Accuracy, F(1/9) = 4.9, p = 0.05, ŋ^2^ = 0.387; Accuracy x Region, F(2/18) = 3.9, p = 0.05, ŋ^2^ = 0.291; Accuracy × Laterality, F(1/9) = 8.9, p = 0.01, ŋ^2^ = 0.479; Accuracy × Region × Laterality, F(2/18) = 14.1, p = 0.001, ŋ^2^ = 0.590). Accordingly, only for right-hand responses were simple Accuracy effects significant at C3 and CP3 electrodes (F(1/9) = 6.02–18.2, p = 0.04 – 0.003, ŋ^2^ = 0.325–0.642).

FCz-PLV peak latency coincided with the moment of response generation and did not depend significantly on response accuracy.

#### DELTA

##### (Delta TOTP (Fig. 5A)

**Figure 5:**
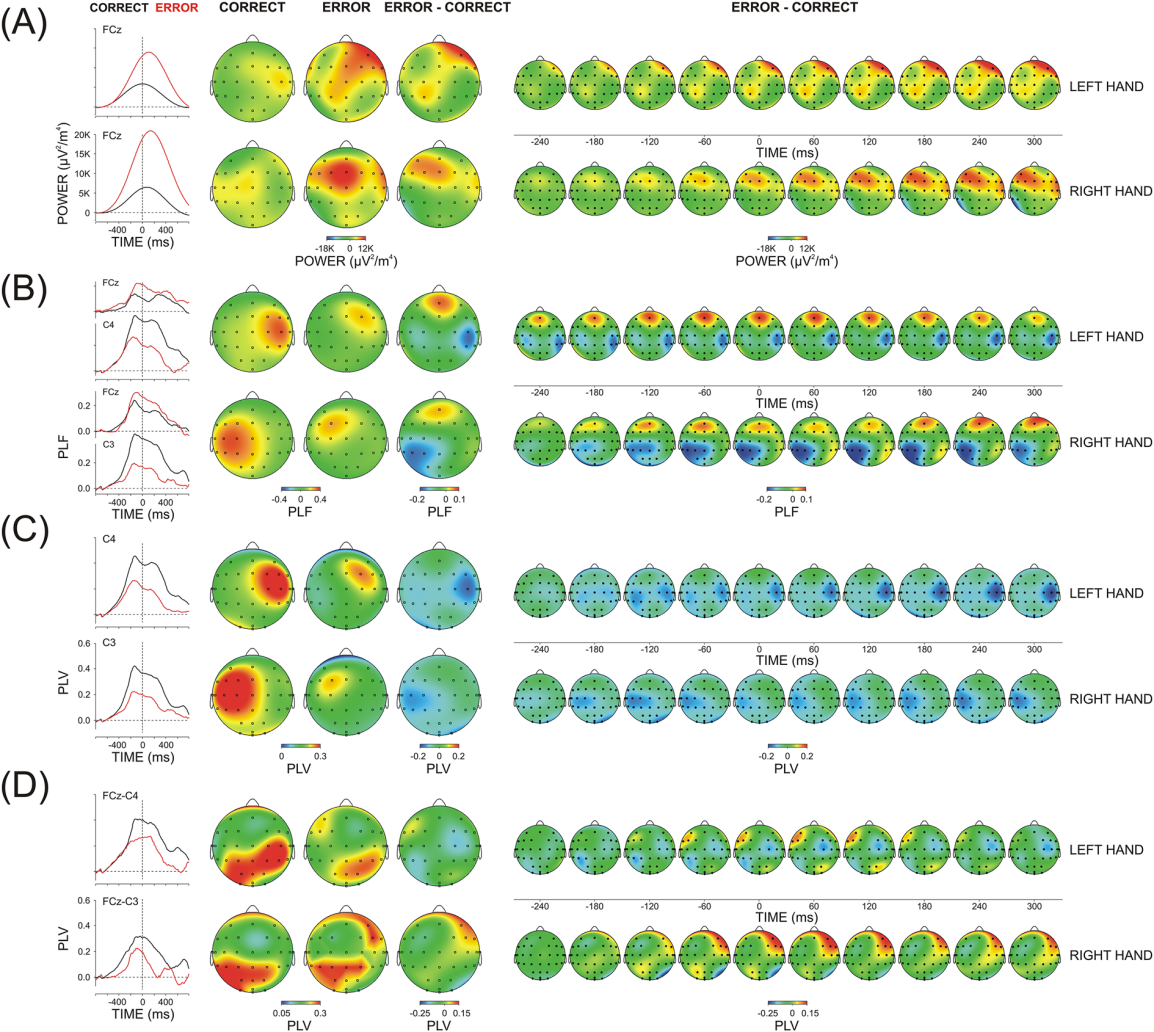
Delta TF component (1–3 Hz) of response-related correct and error potentials elicited by left- and right-hand motor responses (**A**) Total power, (**B**) Temporal synchronization (PLF), (**C**) Region-specific connectedness (regional PLV), (**D**) FCz-guided synchronization FCz-PLV. Left panel – Extracted delta scales at relevant mid-line and contra-lateral electrodes (explanations in the text); Middle panel—Topography maps for correct, error and error minus correct parameter at the time of maximal expression (peak) of the signal at contra-lateral central electrodes the time of maximal expression (signal peak); Right panel—Dynamic topography difference maps (error minus correct). Response onset at 0 ms.

Delta TOTP was larger at the fronto-central and central regions contra-lateral to the response (Laterality, F(2/18) = 5.9/4.7, p = 0.03/0.05, ŋ^2^ = 0.324/0.216; Region (F(2/18) = 6.9/4.9, p = 0.009/0.04, ŋ^2^ = 0.434/0.198 for left- and right-hand responses, respectively).

Delta TOTP was overall larger for errors (Accuracy, F(1/9) = 8.7/9.5, p = 0.015/0.01, ŋ^2^ = 0.492/0.494), which was especially pronounced at fronto-central regions (Accuracy x Region, F(2/18) = 12.1/4.1, p = 0.003/0.05, ŋ^2^ = 0.573/0.175; Accuracy effect at fronto-central regions, F(1/9) = 7.9/21.6, p = 0.02/0.001, ŋ^2^ = 0.380/0.699). Also, error effects were more pronounced at midline and contra-lateral areas (Accuracy × Laterality, F(2/18) = 5.1/5.3, p = 0.04/0.02, ŋ^2^ = 0.175/0.189, Accuracy effect at the midline, F(1/9) = 7.6/7.9, p = 0.02/0.02, ŋ^2^ = 0.381/0.385). Yet, the error-related enhancement of delta TOTP at contra-lateral electrodes reached significance only for right-hand responses (Accuracy effect at the left hemisphere, F(1/9) = 6.9, p = 0.03, ŋ^2^ = 0.433).

Similar to theta, the peak latency of delta TOTP was detected after the response and was delayed for errors (F(1/9) = 10.2, p = 0.01, ŋ^2^ = 0.485, 5 ± 12.3 ms and 28 ± 13.3 ms for correct and error responses – Fig. 5A, left).

##### Delta PLF (Fig. 5B)

Temporal delta synchronization was significantly stronger at the hemisphere contralateral to the response (Laterality, F(2/18) = 40.6/15.8, p < 0.0001/0.001, ŋ^2^ = 0.818/0.788; Region x Laterality, F(4/36) = 6.5/3.2, p = 0.002/0.05, ŋ^2^ = 0.420/0.300).

Errors affected PLF at specific locations: (1) Error-related delta PLF decreased at contra-lateral central/centro-parietal locations. (2) In addition, there was a substantial delta PLF enhancement at the midline frontal/fronto-central electrode (Accuracy x Region, F(2/18) = 9.6/9.5, p = 0.002, ŋ^2^ = 0.516/0.514; Accuracy × Region × Laterality, F(4/36) = 4.2/4.5, p = 0.01, ŋ^2^ = 0.305/0.316). Accordingly, simple Accuracy effects were significant at Fz/FCz (F(1/9) > 9.5, p < 0.01, ŋ^2^ = 0.509/0.534) and contra-lateral C4, CP4/C3,CP3 electrodes (F(1/9) > 6.3, p < 0.03, ŋ^2^ = 0.416–0.517).

Delta PLF peak latency did not depend on whether the response was correct or incorrect (F(1/9) = 3.3, p > 0.05) – Fig. 5B, left panel. It is to be noted that delta PLF manifested two peaks, before and after the response.

Delta PLF was maximal before response production for both correct (− 83.4 ± 10.8 ms) and error responses (− 73 ± 10.5 ms) across regions, and at FCz (− 80 ± 12.5 ms for correct, − 50 ± 11.1 ms for error responses). These observations indicate that the maximal phase stability of motor delta oscillations preceded response generation. Dynamic maps further illustrate that errors induced changes in motor delta PLF as early as 200 ms before the response at both contra-lateral and frontal midline regions.

##### Delta R-PLV (Fig. 5C)

Delta R-PLV was significantly stronger for the hemisphere contralateral to the response (Laterality, F(2/18) = 26.3/12.1, p = 0.0001/0.001, ŋ^2^ = 0.745/0.593).

Errors produced a significant overall decrease in delta R-PLV (Accuracy, F(1/9) = 19.4/8.4, p = 0.002/0.01, ŋ^2^ = 0.684/0.536), which was most pronounced at the contra-lateral central cortex (Region × Laterality × Accuracy, F(2/18) = 3.9/6.8, p = 0.04/0.007, ŋ^2^ = 0.240/0.475; Simple Accuracy effects at contra-lateral C4/C3, F(1/9) > 16.2, p < 0.003, ŋ^2^ = 0.630–0.735).

The maximal peak of delta R-PLV emerged before the response for both correct and error responses (− 80.6 ± 10.1 ms and − 67.2 ± 13.8 ms, respectively). The error-related modulations of delta R-PLV peak were not significant.

##### Delta FCz-PLV (Fig. 5D)

The significant Region × Laterality interaction (F(2/18) = 7.2/7.3, p = 0.005, ŋ^2^ = 0.496/0.501) shows that FCz-guided delta oscillations were most strongly synchronized at the posterior electrodes of the hemisphere contralateral to the response. Maps in Fig. 5D further show that parietal and ipsilateral electrodes were also involved.

As demonstrated by dynamic difference maps (Fig. 5D, right panel) and indicated by Accuracy × Region × Laterality effects (F(2/18) = 6.8/7.1, p = 0.008/0.005, ŋ^2^ = 0.477/0.480), error-related delta FCz-PLV increased at ipsilateral fronto-central regions (simple Accuracy effects at ipsilateral FC3/FC4 (F(1/9) > 6.1, p = 0.02, ŋ^2^ = 0.422/0.432).

The maximal peak of delta FCz-PLV preceded the response for both correct and incorrect reactions (− 82.3 ± 12.1 ms). No significant changes in the peak latency of delta FCz-PLV were found in relation to errors.

## Discussion

The present study was undertaken to analyse oscillatory motor potentials generated during performance errors. It was hypothesized that a wrong or a conflicting motor command would distort the coordination of the motor cortex which would affect motor potentials. Also, the fronto-medial mechanisms of continuous performance monitoring would affect motor networks in the course of incorrect movement generation^13,46,47^. Such mechanisms were expected to produce differences between the oscillatory neurodynamics of correct and error RRPs.

Consistent with this main hypothesis, major results demonstrated that oscillatory activity over extended movement-generating cortical regions differed between correct and incorrect responses. Error-related changes of motor oscillations (1) depended on the frequency, engaging delta and theta frequency bands in specific ways, (2) emerged already before response production, and (3) had specific regional distributions at both posterior sensorimotor and anterior (premotor and medial frontal) areas. Specifically, response-locked phase-stability of delta oscillations at motor and sensorimotor areas contra-lateral to the response was suppressed for incorrect movements. Also, the connectedness of these areas supported by delta networks was substantially reduced during errors. However, this error-related reduction of temporal and spatial synchronization at contra-lateral sensorimotor regions was accompanied by increased phase-stability of motor theta oscillations at bi-lateral premotor regions and by two distinctive error-related patterns at medial frontal regions: (1) a focused enhancement of theta power and (2) an enhancement of phase-stability of delta oscillations.

Consistent with the right handedness of participants, correct responses were faster for the dominant hand. Performance results further showed that errors were slower than correct responses for the faster right hand and were faster than correct responses for the slower left hand. Given that errors were associated with movements with the wrong hand, the lateral asymmetry in error speed can be explained with hand dominance. However, the hemispheric lateralization of movement control mechanisms may be additionally responsible for the observed asymmetry in error performance. It has been shown that in the 4-CRT used here, left-hand responses (in contrast to right-hand responses) are accompanied by motor-related activity at both the contra-lateral (right) and ipsi-lateral (left) motor regions^42^, which may facilitate fast movements with the wrong (right) hand. In addition, previous research has demonstrated the predominant contribution of the right hemisphere to conflict processing^43^ and inhibitory networks functioning^43,48–50^. In contrast, the left premotor and the left parietal cortices are strongly implicated in movement selection and movement attention control^51–53^. These lateralized mechanisms of movement control may underlie the asymmetry of both correct and wrong reactions with the left and the right hand.

In line with previous reports^16–18^, the temporal synchronization of motor delta and theta oscillations manifested a pronounced functional lateralization. They were better synchronized at the hemisphere contralateral to the response side. This functional asymmetry did not depend on the sensory modality and response accuracy. This observation aligns with the notion that delta/theta oscillations play a crucial role in movement generation by either representing a ubiquitous signal from motor neurons^17^ or coordinating motor actions through distributed oscillatory networks^25^.

Importantly, one major effect of errors on RRPs was the reduction of temporal synchronization as reflected by PLF of both delta and theta oscillations at motor/sensorimotor regions contra-lateral to the responding hand. This result demonstrates that error movements are not supported by stable and coherent phases of motor oscillations. Considering the possibility that error-related suppression of temporal synchronization stems from different movement characteristics or errors^17^, the following effects can be expected: (a) error-related alterations of time-domain RRPs reflecting the activation of motor neurons, and (b) differences in the mechanic properties of incorrect and correct movements. However, the time-domain RRPs did not manifest error-related changes before response production (Fig. 2). Also, no differences in the mechanic properties between correct and error movements were obvious at movement initiation before the response (Fig. 1B). In contrast, the suppression of phase-stability appeared to start long before the error movement (Figs. 4B, 5B). Hence, differential activation of movement-generating motor neurons may not be a major source of the error-related suppression of the temporal synchronization of oscillatory responses at contra-lateral regions. Another possible factor compromising the response-locked synchronization during errors may be the simultaneous stimulus/stimulus–response evaluation, onto which motor processes are mapped^46^. Indeed, the post-stimulus processing of conflict between concurrently activated motor programs^10,11^ may have increased the variability in response selection, leading to the unstable alignment of phases as observed here during incorrect responses. In a similar way, dysregulation in transmitting or coordinating motor programs supported by distributed oscillatory networks during cognitive control^25^ may have compromised the stability of oscillatory motor potentials.

Errors also were associated with a decrease of only delta R-PLV which indicates that the spatial connectedness of movement generation regions sub-served by delta networks was especially suppressed during errors. This observation implies that incorrect performance is characterized by a functional disconnection of motor delta networks. Since the presumed functional disconnection appeared long before the wrong movement as indicated by peak latency and implied by dynamic maps (Fig. 5C, left panel), it can be suggested that a decreased communication between motor/sensorimotor regions and other relevant cortical regions may be a specific precursor of errors. It is to be emphasized that the PLV parameter used here may reflect both synchronizations among cortical regions and cortical co-activations induced by sub-cortical sources^39^. Hence, it cannot be excluded that sub-cortical sources involved in movement initiation or coordination such as the basal ganglia or the ACC might have contributed to an impaired co-activation of motor cortical regions during errors^26,54^. Although the source of error-related impairment of motor delta networks cannot be established by current analyses, the results demonstrate that error responses are preceded by a functional disconnection of the motor regions responsible for the generation of the planned movement. Such a functional disconnection may have additionally impaired the temporal phase stability of motor oscillations.

Another major result was that the patterns of error-related desynchronization at posterior (motor/sensorimotor) regions were accompanied by specific oscillatory patterns at anterior (premotor and medial frontal) regions. The anterior patterns were, however, different for delta and theta oscillations. Also, they differed between the phase-locking and power parameters. The temporal synchronization was enhanced by errors (1) at bi-lateral premotor areas for theta oscillations, and (2) at the mid-frontal area for delta oscillations, which was accompanied by (3) an error-related increase in medial fronto-central theta power. Among these three types of anterior error signatures detected here, the medial fronto-central theta power has been the best-established and most intensively studied one (e.g. Refs.^26–28,55^, etc.). Previously, significantly enhanced theta (4–8 Hz) oscillations have been consistently observed over medial-frontal electrodes (centred on FCz) in different sensorimotor conditions in relation to a variety of executive and cognitive control functions—conflict processing, detection of errors, inhibition, performance monitoring, amount of cognitive control, and behavioural re-adjustment^16,56–63^. These reports are consistent with the notion that a theta network “hub” in the medial fronto-central cortex^26,27^ serves to coordinate response execution in different contexts thus supporting various executive functions during cognitive control^25^.

On the other hand, it has been demonstrated that medial theta power is composed of both phase-locked and non-phase-locked oscillations^20,28,64^. According to Cohen and Donner^47^, most of the mid-frontal EEG theta oscillations with behavioural/functional relevance are not phase-locked to the stimulus or to the response and reflect modulation of ongoing theta activity. The present results provide additional strong evidence for the distinction between phase-locked and non-phase-locked midline theta oscillations. They reveal that the error-related enhancement of the phase-locked portion of medial fronto-central theta^16,28,55,65^ is not a local phenomenon, in contrast to total theta energy. Instead, it appears to be a bi-lateral premotor error-related phenomenon. Furthermore, an early engagement of phase-locked theta at ipsilateral areas preceding error response generation was implied by the present results, pointing to a possible involvement of “correct” premotor regions during errors. Whether an excessive temporal synchronization of theta at “correct” premotor areas may reflect the activation of simultaneous competing response options and problems in response selection^51^ remains to be established. Nonetheless, the present results demonstrate that previously detected enhancement of theta phase-locking at FCz during errors represents a localized expression of an error-related synchronized theta signal with a broader distribution at premotor fronto-central regions that may be regarded as a precursor of errors.

In view of the possible broader functional relevance of mid-frontal theta power, the observed here response-locked delta oscillations in the nearby frontal locations may represent a unique error signal at the midline as suggested previously^16^. Recent observations from stimulus-locked potentials have implicated a specific role for frontal medial delta activity in inhibition control^62^. In the present study, only left-hand errors were impulsive (faster than correct responses) and presumably linked to disinhibition (Fig. 1), but no difference between medial frontal delta PLF of left and right-hand errors was detected (Hand effect at Fz and FCz, F(1/9) = 0.3/0.023, p > 0.6). This observation is not fully consistent with a possible role of inhibition for delta PLF expression found here. However, previous research also has suggested that multiple sources in the medial frontal cortex contribute to the expression of error signals at the scalp (e.g. Refs.^6,19,66^). Hence, a unique association between medial frontal delta and errors can be considered in future research in view of the neurodynamics of such multiple sources.

The FCz-guided synchronization during motor response production was maximal at contra-lateral sensorimotor and posterior areas in both the theta and delta bands for both correct and error responses (Figs. 4D, 5D). This result supports the previously reported existence of a connection between medial frontal and movement-generation areas^19,23^. The observation that the FCz-guided synchronization with contra-lateral movement-generating regions was not modulated by errors in the delta range, and was not suppressed for left-hand errors further highlights the suggested specific role of the medial frontal theta in movement monitoring^16^. The reduced theta connections only for the right hand may therefore reflect a functional asymmetry in the monitoring of movements with the right and the left hand or differential monitoring of impulsive vs. delayed incorrect responses^67^.

One limitation of the present study was the small sample size used for analysis which prevents results generalization. Since one major methodological goal was to evaluate error signals in a reliable way by controlling for signal-to-noise ratio, only subjects with a sufficient number of artefact-free error trials were used. This approach considered mainly technical artefacts and not the actual number of performance errors. Hence, the included sample was not biased by performance quality and guaranteed the assessment of error potentials. As another limitation, the precise temporal evolution of motor oscillatory parameters was not analysed, and accordingly, establishing which of the novel motor correlates of error processing may be regarded as precursors of errors or as signals sub-serving evaluative post-error processes needs further exploration.

## Conclusion

Together, these observations indicate that the electrophysiological signatures of performance errors are not limited to the medial frontal signals. They demonstrate a decrease in connectivity of delta networks at contra-lateral motor/sensorimotor regions and a bilateral enhancement of phase-stability of motor theta oscillations at premotor regions before and during error generation that may serve as precursors of incorrect movements. The present results also provide a refined picture of the medial frontal processes established previously in relation to error processing in humans. Hence, novel correlates of error processing in the brain involving the dynamics of oscillatory motor networks at extended cortical regions are revealed. New directions of research can be envisaged to study the precise functional relevance of these neurophysiological signatures for movement generation, control, and monitoring.

## Data Availability

The datasets used and analysed during the current study are available from the corresponding author on reasonable request.
